# Incidence and associated risk factors for premature death in the Tehran Lipid and Glucose Study cohort, Iran

**DOI:** 10.1186/s12889-019-7056-y

**Published:** 2019-06-10

**Authors:** Ali Eslami, Seyed Sina Naghibi Irvani, Azra Ramezankhani, Nazanin Fekri, Keyvan Asadi, Fereidoun Azizi, Farzad Hadaegh

**Affiliations:** 1 Prevention of Metabolic Disorders Research Center, Research Institute for Endocrine Sciences, Shahid Beheshti University of Medical Sciences, Floor 3th, Number 24, Yemen Street, ShahidChamran Highway, P.O. Box: 19395-4763, Tehran, Iran; 2 Department of Epidemiology and Biostatistics, School of Public Health, Tehran University of Medical Sciences, Tehran, Islamic Republic of Iran; 3 Endocrine Research Center, Research Institute for Endocrine Sciences, Shahid Beheshti University of Medical Sciences, Tehran, Iran

**Keywords:** Premature mortality, Risk factors, Cohort study, Population attributable fractions, Incidence

## Abstract

**Background:**

The incidence and associated risk factors for premature death were investigated in a population-based cohort study in Iran.

**Methods:**

A total of 7245 participants (3216 men), aged 30–70 years, were included. We conducted Cox proportional hazards models to identify the risk factors for premature death. For each risk factor, hazard ratio (HR), 95% confidence intervals (95% CI) and population attributable fraction (PAF) were calculated.

**Results:**

After a median follow-up of 13.8 years, 262 premature deaths (153 in men) occurred. Underlying causes of premature deaths were cardiovascular disease (CVD) (*n* = 126), cancer (*n* = 51), road injuries (*n* = 15), sepsis and pneumonia (*n* = 9) and miscellaneous reasons (*n* = 61). The age-standardized incident rate of premature death was 2.35 per 1000 person years based on WHO standard population. Hypertension [HR 1.40, 95% CI (1.07–1.83)], diabetes (2.53, 1.94–3.29) and current smoking (1.58, 1.16–2.17) were significant risk factors for premature mortality; corresponding PAFs were 12.3, 22.4 and 9.2%, respectively. Overweight (body mass index (BMI): 25–29.9 kg/m^2^) (0.65, 0.49–0.87) and obesity (BMI ≥30 kg/m^2^) (0.67, 0.48–0.94) were associated with decreased premature mortality. After replacing general adiposity with central adiposity, we found no significant risk for the latter (0.92, 0.71–1.18). Moreover, when we excluded current smokers, those with prevalent cancer/cardiovascular disease and those with survival of less than 3 years, the inverse association between overweight (0.59, 0.39–0.88) and obesity (0.67, 0.43–1.04), generally remained unchanged; although, diabetes still showed a significant risk (2.62, 1.84–3.72).

**Conclusions:**

Controlling three modifiable risk factors including diabetes, hypertension and smoking might potentially reduce mortality events by over 40%, and among these, prevention of diabetes should be prioritized to decrease burden of events. We didn’t confirm a negative impact of overweight and obesity status on premature mortality events.

## Background

Globally 42% of all deaths caused by non-communicable diseases (NCD) occurred before the age of 70 years and this rate ranges from 28% in high income countries to 48% in low- and middle-income countries. Based on World Health Organization (WHO) estimates, total NCD deaths will escalate to 52 million in 2030 [1]. Hence, the global action plan offers a paradigm shift and, if implemented completely, will attain 9 voluntary global targets, including the overarching target of 25% relative reduction in premature mortality from NCD by 2025 [2].

Among the traditional risk factors for premature mortality, smoking was shown to be the predominant risk factor for premature mortality; although, obesity, hypertension and poor diet also showed substantial additional impacts [3]. However, the effect of body mass index (BMI) categories, on premature mortality is not consistent among different studies [4, 5].

Recently we showed that incident premature cardiovascular disease (CVD) was 5.06 per 1000 person-years in a Tehranian population [6]. In our current study among Iranian adults in a population-based cohort of the Tehran Lipid and Glucose Study (TLGS), we extended our previous research to examine the incidence of premature all-cause mortality and associated risk factors and to determine the corresponding population attributable fractions (PAF), defined as a proportion of the outcome in the population that could be reduced, if the exposure were potentially eliminated [7]. As a second goal, we evaluated the impact of obesity categories on mortality after limiting confounders by excluding current smokers, participants with a chronic disease and those with survival of less than 3 years.

## Methods

### Study subjects

Details of the study design and sampling frame of the TLGS have been previously published. Briefly, a representative sample (*n* = 18,555) of Tehranian participants aged ≥3 years were recruited during the first (1999–2001) and second phase (2002–2005) [8]. According the WHO definition of premature mortality [1], 9030 participants (5026 women) aged 30–69 years at baseline were included. Participants without any follow-up data (*n* = 818) and with missing data on study variables (*n* = 967) were excluded. The remaining 7245 participants (80.23% of eligible sample) were followed up until end of this study (20 March 2014).

### Baseline data collection

Information on demographic characteristics, medication use, medical history, family history, and use of tobacco products was collected at baseline using a standardized questionnaire [8]. A detailed description of anthropometric, clinical and laboratory measurements have been reported elsewhere [8].

### Definition of terms and outcome variable

*Definition of risk factors has been shown in* Table 1*.* The study outcome was time to death from any cause. Mortality data was gathered by the trained nurses through the annual follow-up of participants. All deaths were confirmed with death certificate data, medical records of the hospitalized patients, forensic medicine report and verbal autopsy if needed. The collected data were also confirmed by a committee included expert specialists [8].

**Table 1:** Definition of different premature cardiovascular disease risk factors; Tehran Lipid and Glucose study (1999–2014)

Risk factor	Definition
BMI	
Normal	BMI < 25 kg/m^2^
Overweight	25 ≤ BMI < 30 kg/m^2^
Obese	BMI ≥30 kg/m^2^
Central obesity	WC ≥95 cm [9]
Hypercholesterolemia	TC ≥ 6.21 mmol/L or using lipid lowering drugs
Hypertension	SBP ≥140 mmHg or DBP ≥90 mmHg or using any high blood pressure medications
Diabetes	FPG ≥7 mmol/L, or 2 h-PG ≥11.1 mmol/L or taking anti-diabetic drugs
Low physical activity	• Doing exercise or labor < 3 times a week (in first phase) • Achieving < 600 MET (metabolic equivalent task)-minutes per week (in second phase)
Current smoker	Participants who used cigarettes or other smoking implements daily, non-daily and occasionally at baseline
Education	
Illiterate/primary	0–5 years of schooling
Cycle/diploma	6–12 years of schooling
Higher than diploma	> 12 years of schooling
FH-CVD	*Prior diagnosis of CVD in a* male first degree relative < 55 years or in a female first degree relative < 65 years

*BMI* body mass index, *FPG* fasting plasma glucose, *2-h PG* 2-h post load glucose test, *TC* total cholesterol, *SBP* systolic blood pressure, *DBP* diastolic blood pressure, *FH-CVD* family history of premature cardiovascular disease, *WC* waist circumference

### Statistical methods

We estimated the annualized incidence of premature deaths (95% confidence intervals (CI)) per 1000 person-years. The age-standardized incidence rates were obtained according WHO standard population and total population of Iran at 2016 census [10].

The date of mortality from any cause was considered as the event date. Time to event was calculated as the difference between the entered study date and the date of death up to 20 March 2014, when follow-up ceased. We censored individuals who were lost to follow-up, who were still alive at the end of follow-up period or who reaching the age of 70 years.

Hazard ratios (HRs) for each of the risk factors were estimated using the Cox proportional hazards models considering the age as time scale, which refers to using the date of birth as a starting point, and utilize age as the time-to-event [11]. We used the scaled Schoenfeld residuals to check the proportional hazards assumption in the multivariable Cox models. For all proportional hazards models, proportionality assumptions were met. We found no interactions between sex and different risk factors (all *p* values > 0.05); hence, the analysis was performed in the pooled samples to reach full statistical power. The effects of waist circumference (WC) and BMI were analyzed in different multivariable Cox models. The following formula was used for the calculation of PAF [7]:

P × [(HR_adj_ – 1)/HR_adj_] × 100,

In the above formula, “P” indicates the prevalence of the risk factors among participants in whom premature deaths were occurred, and HR_adj_ shows the multivariable adjusted HR for each risk factor.

Statistical analyses were conducted using Stata 12.0 (StataCorp LP, TX, USA), and all *P* values < 0.05 were considered as significant.

## Results

Baseline characteristics of participants are shown in Table 2. The mean (standard deviation (SD)) age of study population (*n* = 7245) was 46.08 (11.04) years at baseline. The prevalence of overweight, obesity, central obesity, hypercholesterolemia, hypertension, diabetes, low physical activity and current smokers were 43.6, 27.4, 37.3, 28.3, 25.7, 13.9, 71.6 and 16.5%, respectively (Table 2).

**Table 2:** Baseline characteristics of participants; Tehran Lipid and Glucose study (1999–2014)

Continuous variables	Mean (SD)
Age (years)	46.08 (11.04)
BMI (kg/m^2^)	27.62 (4.56)
WC (cm)	90.85 (11.48)
FPG (mmol/L)	5.62 (1.98)
2-h PG (mmol/L)	6.79 (3.25)
TC (mmol/L)	5.56 (1.19)
SBP (mmHg)	121.14 (19.44)
DBP (mmHg)	78.89 (10.97)
Categorical variables	Frequency (%)
BMI	
Normal (< 25 kg/m^2^)	2104 (29.0)
Overweight (25–30 kg/m^2^)	3158 (43.6)
Obese (≥30 kg/m^2^)	1981 (27.4)
Central obesity	2704 (37.3)
Hypercholesterolemia	2050 (28.3)
Hypertension	1860 (25.7)
Diabetes	1008 (13.9)
Low physical activity	5183 (71.6)
Current smoker	1196 (16.5)
Education	
Illiterate/primary	2888 (39.9)
Cycle/diploma	2466 (47.9)
Higher than diploma	889 (12.3)
FH-CVD	1228 (17.0)
Prevalent CVD	390 (5.4)

Data are shown as mean (SD) or frequency (%)

*BMI* body mass index, *WC* waist circumference, *FPG* fasting plasma glucose, *2-h PG* 2-h post load glucose test, *TC* total cholesterol, *SBP* systolic blood pressure, *DBP* diastolic blood pressure, *FH-CVD* family history of premature cardiovascular disease

Central obesity defined as waist circumference ≥ 95 cm; Hypercholesterolemia was defined as serum TC ≥6.21 mmol/L or using lipid lowering drugs; Hypertension was defined as SBP ≥140 mmHg or DBP ≥90 mmHg or under drug treatment; Diabetes was defined as FPG ≥7 mmol/L or 2-h PG ≥11.1 mmol/L or taking anti-diabetic medication; Low physical activity was defined as participating in vigorous physical activity less than three days per week. Those participants who entered in the second phase, were considered low physically active when achieving less than 600 MET (metabolic equivalent task)-minutes per week; FH-CVD was defined as diagnosis of CVD in male or female first-degree blood relatives, aged < 55 years or aged < 65 years old, respectively

At a median follow-up of 13.84 years (inter-quartile range: 9.29–14.43 years), 262 premature deaths (153 in men and 109 in women) occurred. The highest proportion of premature deaths was due to CVD (48%). Cancer accounted for about 19% of the premature deaths, and the remaining deaths were related to sepsis and pneumonia (3%), accidents (6%) and miscellaneous reasons (23%).

The crude incidence rate of premature death (95% CI) was 3.15 (2.79–3.55) per 1000 person-years. The age-standardized incidence rates were 1.90 (1.68–2.14) and 2.35 (2.07–2.64) per 1000 person-years according to the Iranian and WHO standard population, respectively.

Table 3 shows the uni-variable contribution of each candidate predictor to the risk of premature mortality. Being female, overweight and obesity were negative risk factor for premature mortality. However, hypertension, diabetes, smoking, family history of premature CVD and prevalent CVD were positive risk factor for premature mortality.

**Table 3:** Hazard ratios from the uni-variable analysis of potential risk factors in relation to premature mortality; Tehran Lipid and Glucose study (1999–2014)

	Hazard ratio (95% CI)	*P* value
Sex (Female)	0.55 (0.43–0.71)	< 0.001
Body mass index		
Normal	Reference	
Overweight	0.67 (0.51–0.90)	0.007
Obese	0.66 (0.49–0.91)	0.011
Hypercholesterolemia	1.14 (0.89–1.46)	0.308
Hypertension	1.47 (1.14–1.90)	0.003
Diabetes	2.62 (2.03–3.39)	< 0.001
Smoking	1.89 (1.41–2.53)	< 0.001
Education		
Illiterate/primary	Reference	
Cycle/diploma	1.09 (0.83–1.44)	0.529
Higher than diploma	0.94 (0.59–1.50)	0.797
Low physical activity	1.16 (0.88–1.54)	0.293
FH-CVD	1.45 (1.10–1.95)	0.009
Prevalent CVD	2.86 (2.08–3.92)	< 0.001

The analysis was performed using the Cox proportional hazards models considering the age as time scale

All definitions and variables explained as footnotes for Table 2

CI: confidence interval

In multi-variable model (Table 4), being female, overweight and obesity were associated with decreased risk of premature deaths with the following HRs (95% CI): 0.65 (0.49–0.87), 0.67 (0.48–0.94) and 0.57 (0.43–0.76), respectively. However, hypertension, diabetes, smoking, family history of premature CVD and prevalent CVD were associated with increased risk of premature deaths [1.40 (1.07–1.83), 2.53 (1.94–3.29) and 1.58 (1.16–2.17), 1.43 (1.07–1.91) and 2.18 (1.57–3.02), respectively]; the PAFs were 12.3, 22.4, 9.2, 6.9 and 10.3%, respectively. When we replaced BMI categories with abdominal obesity, as defined by high WC, the results remained essentially unchanged and the presence of abdominal obesity was not associated with risk of premature death [0.92 (0.71–1.18)] (data not shown).

**Table 4:** Hazard ratios and population attributable fraction from the multi-variable analysis of potential risk factors in relation to premature mortality; Tehran Lipid and Glucose study (1999–2014)

	Hazard ratio (95% CI)	*P* value	Prevalence^a^	PAF, %^b^
Sex (Female)	0.57 (0.43–0.76)	< 0.001	0.42 (0.36–0.47)	−31.7
Body mass index				
Normal	Reference		0.33 (0.28–0.39)	
Overweight	0.65 (0.49–0.87)	0.004	0.39 (0.33–0.45)	−21.0
Obese	0.67 (0.48–0.94)	0.020	0.28 (0.22–0.33)	−13.8
Hypercholesterolemia	1.13 (0.87–1.46)	0.377	0.40 (0.35–0.46)	
Hypertension	1.40 (1.07–1.83)	0.016	0.43 (0.37–0.48)	12.3
Diabetes	2.53 (1.94–3.29)	< 0.001	0.37 (0.31–0.43)	22.4
Smoking	1.58 (1.16–2.17)	0.004	0.25 (0.20–0.30)	9.2
Education				
Primary/Illiterate	Reference		0.56 (0.50–0.61)	
Cycle/Diploma	0.89 (0.67–1.19)	0.445	0.37 (0.31–0.42)	
Higher than diploma	0.79 (0.48–1.28)	0.329	0.08 (0.05–0.11)	
Low physical activity	1.10 (0.83–1.47)	0.491	0.25 (0.20–0.30)	
FH-CVD	1.43 (1.07–1.91)	0.015	0.23 (0.18–0.28)	6.9
Prevalent CVD	2.18 (1.57–3.02)	< 0.001	0.19 (0.14–0.23)	10.3

The analysis was performed using the Cox proportional hazards models considering the age as time scale

All definitions and variables explained as footnotes for Table 2

^a^Prevalence and CI of the risk factors among individuals who had an event

^b^PAF: population attributable fraction calculated for *p* value< 0.05; formula for calculating PAF = P×[(HR_adj_–1)/HR_adj_] × 100

*CI* confidence interval, *P* prevalence

In a sensitivity analysis, as recently suggested by other researchers [5, 12], to address the reverse causality impact of chronic disease and current smoking as well as those with survival of less than 3 years, we repeated our data analysis among a 5442 non-smoker population, without prevalent CVD/cancer who had survived at least 3 years. As shown in Table 5, being overweight [0.59 (0.39–0.88)] was still associated with lower risk for premature mortality. There was also a marginally significant association between being obese [0.67 (0.43–1.04), *P* = 0.072] and lower risk for premature mortality. Furthermore, the presence of diabetes [2.62 (1.84–3.72)] was also associated with increased risk of premature death.

**Table 5:** Hazard Ratios and population attributable fraction from the multi-variable analysis of potential risk factors in relation to premature mortality excluded current smokers, prevalent CVD/cancer and survival less than 3 years; Tehran Lipid and Glucose study (1999–2014)

	Hazard ratio (95% CI)	*P* value	Prevalence^a^	PAF, %^b^
Sex (Female)	0.67 (0.46–0.97)	0.036	0.55 (0.47–0.63)	−27.1
Body mass index				
Normal	Reference		0.30 (0.23–0.38)	
Overweight	0.59 (0.39–0.88)	0.011	0.36 (0.29–0.44)	−25.0
Obese	0.67 (0.43–1.04)	0.072	0.33 (0.26–0.41)	−16.3
Hypercholesterolemia,	1.05 (0.74–1.50)	0.781	0.38 (0.30–0.46)	
Hypertension	1.12 (0.78–1.60)	0.536	0.40 (0.31–0.47)	
Diabetes	2.62 (1.84–3.72)	< 0.001	0.37 (0.30–0.45)	22.9
Education				
Primary/Illiterate	Reference		0.58 (0.50–0.66)	
Cycle/Diploma	0.98 (0.66–1.46)	0.923	0.32 (0.24–0.39)	
Higher than diploma	1.17 (0.64–2.11)	0.611	0.10 (0.05–0.15)	
Low physical activity	1.35 (0.91–2.00)	0.139	0.23 (0.17–0.30)	
FH-CVD	1.58 (1.07–2.32)	0.022	0.23 (0.16–0.30)	8.4

All definitions and variables explained as footnotes for Table 2

^a^Prevalence and CI of the risk factors among individuals who had an event

^b^PAF: population attributable fraction calculated for *p* value< 0.05; formula for calculating PAF=P×[(HR_adj_ 1)/HR_adj_] × 100

*CI* confidence interval, *CVD* cardiovascular disease, *P* prevalence

## Discussion

Our study showed that the three major modifiable risk factors including diabetes, hypertension and current smoking, altogether, account for over 40% of premature deaths. Among them, the highest PAF was attributable to diabetes, the value of which was over two-fold that of prevalent CVD. Also, overweight and obesity were associated with a lower risk, even among non-smokers without prevalent CVD/cancer who had survived at least 3 years.

Our finding of diabetes, hypertension and smoking being the independent risk factors of premature mortality, largely support previous findings [3]. Although some previous studies, using case-control or cross-sectional design, conducted among Middle-East populations [13, 14], have investigated the impact of different risk factors and mortality events, to the best of our knowledge, none have been conducted using a population-based cohort study design.

It is well known that premature deaths are higher in people with diabetes than those without diabetes [15], a risk higher for younger diabetics than for older ones [16]. We observed that diabetes accounted for more than 22% of premature death. Interestingly, in line with previous studies [17, 18], our analysis demonstrated that in terms of PAF, diabetes results in more than two-fold risk for premature deaths than prevalent CVD.

In this study, when we excluded current smokers, prevalent CVD/cancer as well as those with survival of less than 3 years, the presence of diabetes still showed about a three-fold [2.62 (1.84–3.72)] risk for premature mortality events. It has been shown that over 470,000 Iranian adults are currently unaware of their disease [19]. Hence, prevention, as well as earlier identification and providing appropriate control for diabetic patients, should be the first priority for controlling premature deaths, even among non-smokers.

The high prevalence [20] and incidence [21] of hypertension have been reported among Iranian populations before. Recently, we found among a middle-aged population, uncontrolled hypertension had about a three-fold risk for all-cause mortality events [22]. Our analyses showed about 12% of premature deaths were attributable to hypertension. There is much evidence showing high salt intake, among Iranian population, might potentially be the main risk factor for hypertension, with a decrease in salt intake leading to a decrease in blood pressure (BP) [23]. Therefore, health policy makers in our country should focus on two strategic concepts; first, early detection of unfavorable BP levels and early treatment aiming at returning BP to below 140/90 mmHg; second, lifestyle modification especially reduction of dietary salt intake as an effective primary approach for prevention of hypertension.

Unfortunately, the current studies have shown an increasing trend in smoking among Tehranian adults [24, 25]. In our study, quitting smoking can prevent over 9% of premature mortality events. Muller et al. [3] showed smoking was the most powerful risk factor for premature mortality in Europe. However, in our study the PAF for smoking was lower than those of diabetes and hypertension. It is estimated that 70% increase in tobacco price, would avoid one-quarter of all expected premature deaths from tobacco. Data also shows that tobacco is 70% cheaper in low and middle income countries, than in high income ones [26]. Hence, the strategies of policy makers should focus on increasing tobacco taxes, increasing population awareness, and establishing bans on smoking in public places.

Our data showed that in both univariate and multivariate analysis, being overweight and obese were associated with lower risk for premature mortality, a phenomena called the “obesity paradox” [27]. This is in line with finding from a meta-analysis of 97 studies with 270,000 deaths among 2.88 million participants, which supports the protective effects of being overweight on all-cause mortality [4]. However, the Global BMI Mortality Collaboration reported a hazardous effect of being overweight and obese on all-cause mortality by restricting analysis to never smokers and excluding pre-existing chronic disease and the mortality during the first period of follow-up [5]. In our data analysis, using the approach suggested by the Global BMI mortality collaboration [5], we didn’t find a hazardous impact for obesity categories. In fact, being overweight showed a 41% lower risk of premature mortality events, even in the presence of obesity mediators, i.e., diabetes, hypertension and hypercholesterolemia. Interestingly, when we replaced general adiposity (BMI) with central adiposity (WC), we again found no positive risk for mortality events. Hence, we speculate a protective effect of moderately increased body fat among Iranian individuals; which might be attributable to earlier presentation of obese individuals, greater probability of seeking and receiving medical treatment, and benefits of higher metabolic reserves [4].

The strength of this study is the use of the TLGS as a large population-based-cohort with a long follow-up data with reliable surveillance for death events and use of actual measurements of variables rather than self-reported data.

Our study has some limitations; first, due to low incidence of premature death among participants, our sample size might lack sufficient statistical power to detect risk factors in a sex stratified manner or according to the specific cause of death. Second, as inherent to any prospective study, the level of risk factors was assessed only at the baseline examination and a possible change during the follow-up was not considered in our data analysis. As such, some degree of misclassification might have occurred, leading to the attenuation of the hazard ratios for different risk factors. Third, regarding socio-economic status of the study population we assessed only educational status and did not evaluate certain other important components such as income status and the accurate occupational position of study population. Last but not least perhaps, this study was conducted among Tehranian adult population, so our results cannot be extrapolated to the whole country or other Middle-Eastern populations.

## Conclusion

In the current study we showed an age-standardized incidence rate of 2.35 per 1000 person-years based on WHO standard population for premature mortality during more than a decade of follow-up. Controlling three modifiable risk factors including diabetes, hypertension and smoking could potentially reduce mortality events by over 40%, viz. the prevention and control of diabetes being prioritized by national policy makers to decrease the burden of premature mortality events. We didn’t confirm a negative impact of overweight and obesity on premature mortality among our populations.

## Data Availability

The datasets used and/or analysed during the current study are available from the corresponding author on reasonable request.

## Abbreviations

2-h PG: 2-h post load glucose test
BMI: Body mass index
CI: Confidence intervals
CVD: Cardiovascular disease
DBP: Diastolic blood pressure
FPG: Fasting plasma glucose
HR: Hazard ratio
MET: Metabolic equivalent task
NCD: Non-communicable diseases
PAFs: Population attributable fractions
SBP: Systolic blood pressure
SD: Standard deviation
TC: Total cholesterol
TLGS: Tehran Lipid and Glucose Study
WC: Waist circumference
WHO: World Health Organization

## References

[1] Mendis S, Armstrong T, Bettcher D, Branca F, Lauer J, Mace C, et al. Global status report on noncommunicable diseases 2014: World Health Organization; 2015.

[2] Mendis S (2017). Global progress in prevention of cardiovascular disease. Cardiovasc Diagn Ther.

[3] Muller DC, Murphy N, Johansson M, Ferrari P, Tsilidis KK, Boutron-Ruault M-C (2016). Modifiable causes of premature death in middle-age in Western Europe: results from the EPIC cohort study. BMC Med.

[4] Flegal KM, Kit BK, Orpana H, Graubard BI (2013). Association of all-cause mortality with overweight and obesity using standard body mass index categories: a systematic review and meta-analysis. JAMA..

[5] Collaboration GBM (2016). Body-mass index and all-cause mortality: individual-participant-data meta-analysis of 239 prospective studies in four continents. Lancet..

[6] Eslami A, Mozaffary A, Derakhshan A, Azizi F, Khalili D, Hadaegh F (2017). Sex-specific incidence rates and risk factors of premature cardiovascular disease. A long term follow up of the Tehran lipid and glucose study. Int J Cardiol.

[7] Rockhill B, Newman B, Weinberg C (1998). Use and misuse of population attributable fractions. Am J Public Health.

[8] Azizi F, Ghanbarian A, Momenan AA, Hadaegh F, Mirmiran P, Hedayati M (2009). Prevention of non-communicable disease in a population in nutrition transition: Tehran lipid and glucose study phase II. Trials..

[9] Hadaegh F, Zabetian A, Sarbakhsh P, Khalili D, James W, Azizi F (2009). Appropriate cutoff values of anthropometric variables to predict cardiovascular outcomes: 7.6 years follow-up in an Iranian population. Int J Obes.

[10] Ahmad OB, Boschi-Pinto C, Lopez AD, Murray CJ, Lozano R, Inoue M (2001). Age standardization of rates: a new WHO standard: World Health Organization Geneva.

[11] Pencina MJ, Larson MG, D'Agostino RB (2007). Choice of time scale and its effect on significance of predictors in longitudinal studies. Stat Med.

[12] Collaborators G (2017). Health effects of overweight and obesity in 195 countries over 25 years. N Engl J Med.

[13] Tokuç B, Ayhan S, Saraçoğlu GV (2016). The burden of premature mortality in Turkey in 2001 and 2008. Balkan Med J.

[14] Mokdad AH, Jaber S, Aziz MIA, AlBuhairan F, AlGhaithi A, AlHamad NM (2014). The state of health in the Arab world, 1990–2010: an analysis of the burden of diseases, injuries, and risk factors. Lancet..

[15] Mulnier H, Seaman H, Raleigh V, Soedamah-Muthu S, Colhoun H, Lawrenson R (2006). Mortality in people with type 2 diabetes in the UK. Diabet Med.

[16] Kerr D, Partridge H, Knott J, Thomas P (2011). HbA1c 3 months after diagnosis predicts premature mortality in patients with new onset type 2 diabetes. Diabet Med.

[17] Bozorgmanesh M, Hadaegh F, Sheikholeslami F, Ghanbarian A, Azizi F (2012). Shadow of diabetes over cardiovascular disease: comparative quantification of population-attributable all-cause and cardiovascular mortality. Cardiovasc Diabetol.

[18] Nwaneri C, Cooper H, Bowen-Jones D (2013). Mortality in type 2 diabetes mellitus: magnitude of the evidence from a systematic review and meta-analysis. The Br J Diab Vasc Dis.

[19] Esteghamati A, Etemad K, Koohpayehzadeh J, Abbasi M, Meysamie A, Noshad S (2014). Trends in the prevalence of diabetes and impaired fasting glucose in association with obesity in Iran: 2005–2011. Diabetes Res Clin Pract.

[20] Esteghamati A, Abbasi M, Alikhani S, Gouya MM, Delavari A, Shishehbor MH (2008). Prevalence, awareness, treatment, and risk factors associated with hypertension in the Iranian population: the national survey of risk factors for noncommunicable diseases of Iran. Am J Hypertens.

[21] Bozorgmanesh M, Hadaegh F, Mehrabi Y, Azizi F (2011). A point-score system superior to blood pressure measures alone for predicting incident hypertension: Tehran lipid and glucose study. J Hypertens.

[22] Lotfaliany M, Akbarpour S, Mozafary A, Boloukat RR, Azizi F, Hadaegh F (2015). Hypertension phenotypes and incident cardiovascular disease and mortality events in a decade follow-up of a Middle East cohort. J Hypertens.

[23] Shahram Rafieifar M, Hossein Kazemeini M (2016). Strategies and opportunities ahead to reduce salt intake. Arch Iran Med.

[24] Peto R, Lopez A, Pan H, Boreham J, Thun M. Mortality from smoking in developed countries, 1950-2020: trends in smoking-attributed mortality and Total. Mortality. 2015.

[25] Jahangiri-Noudeh Y, Akbarpour S, Lotfaliany M, Zafari N, Khalili D, Tohidi M (2014). Trends in cardiovascular disease risk factors in people with and without diabetes mellitus: a middle eastern cohort study. PLoS One.

[26] Jha P (2009). Avoidable global cancer deaths and total deaths from smoking. Nat Rev Cancer.

[27] Banack HR, Kaufman JS (2014). The obesity paradox: understanding the effect of obesity on mortality among individuals with cardiovascular disease. Prev Med.

